# Correction: Evaluation of a Prednisolone Acetate-Loaded Subconjunctival Implant for the Treatment of Recurrent Uveitis in a Rabbit Model

**DOI:** 10.1371/journal.pone.0105658

**Published:** 2014-08-11

**Authors:** The PLOS ONE Staff

The second author’s name is spelled incorrectly. The correct name is: Xu Wen Ng.
